# Supplementary material for: A proteome-wide structural systems approach reveals insights into protein families of all human herpesviruses
Source: Nat Commun. 2024 Nov 26;15:10230. doi: 10.1038/s41467-024-54668-2 (PMC11599850; doi:10.1038/s41467-024-54668-2)
Supplement: Supplementary file 1 — Supplementary Information [file 41467_2024_54668_MOESM1_ESM.pdf]

A proteome-wide structural systems approach reveals insights into protein families of all human herpesviruses

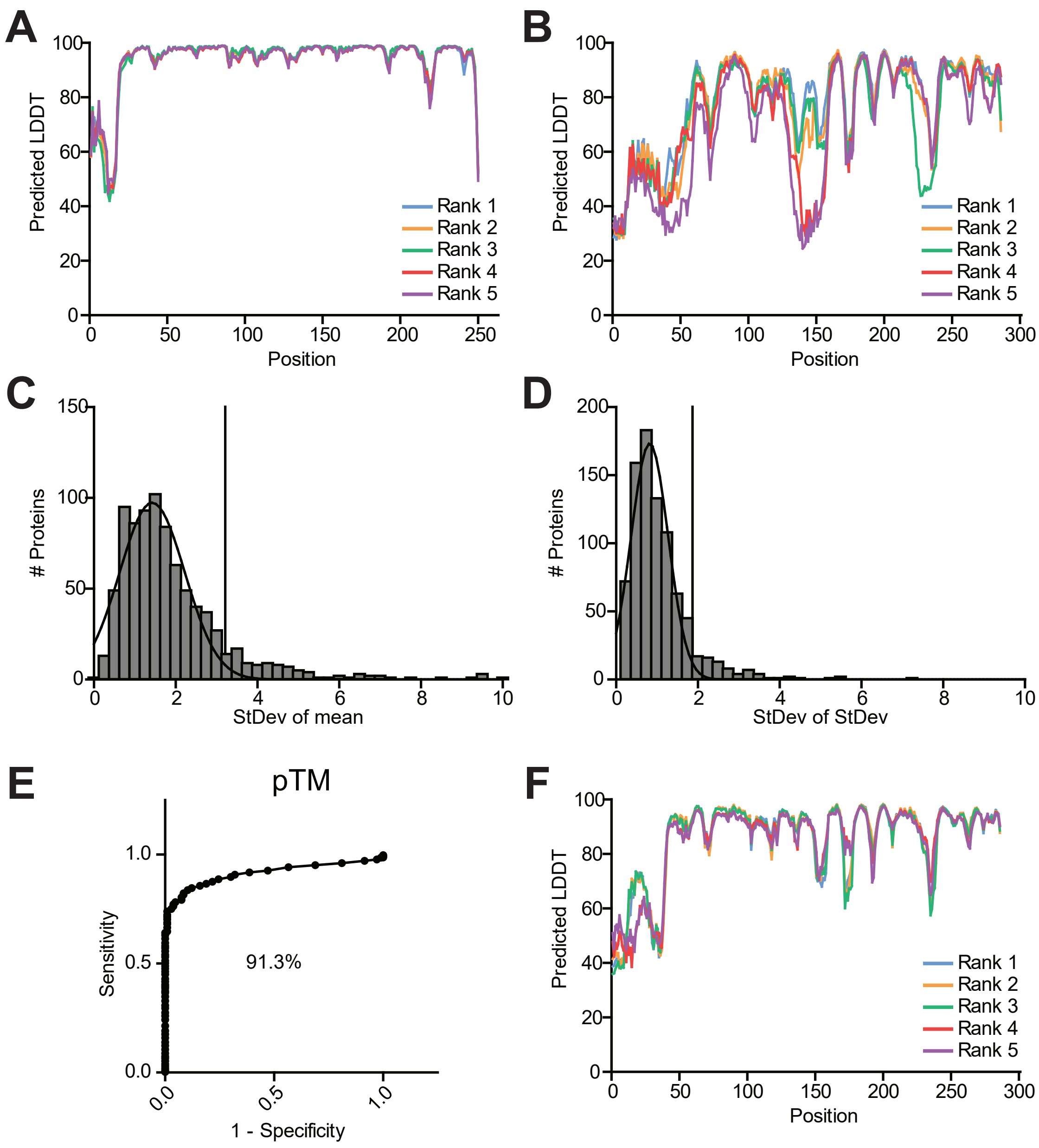

Supplementary Fig. 1. **Developing thresholds for evaluating model quality.** A. pLDDT plot of HCMV UL114 as an example of a protein where the pLDDT curves of the 5 models are consistent. B. pLDDT plot of HHV-7 U29 as an example of a protein where the pLDDT curves of the 5 models are inconsistent. C. Histogram of the standard deviation (StDev) of the mean of the pLDDT for each model for all LocalColabFold models. The threshold (black vertical bar) is set at the mean + 2x StDev, 3.2. D. Histogram of the StDev of the StDev of the pLDDT for each model for all LocalColabFold models. The threshold (black vertical bar) is set at the mean + 2x StDev, 1.9. E. ROC analysis of the pTM of all LocalColabFold models that passed the quality scores versus a negative control harboring scrambled amino acid sequences. The area under the curve is 91.3%. F. pLDDT curve of HHV-7 U29 as an example of a protein that passed the quality score thresholds after AlphaFold was used.

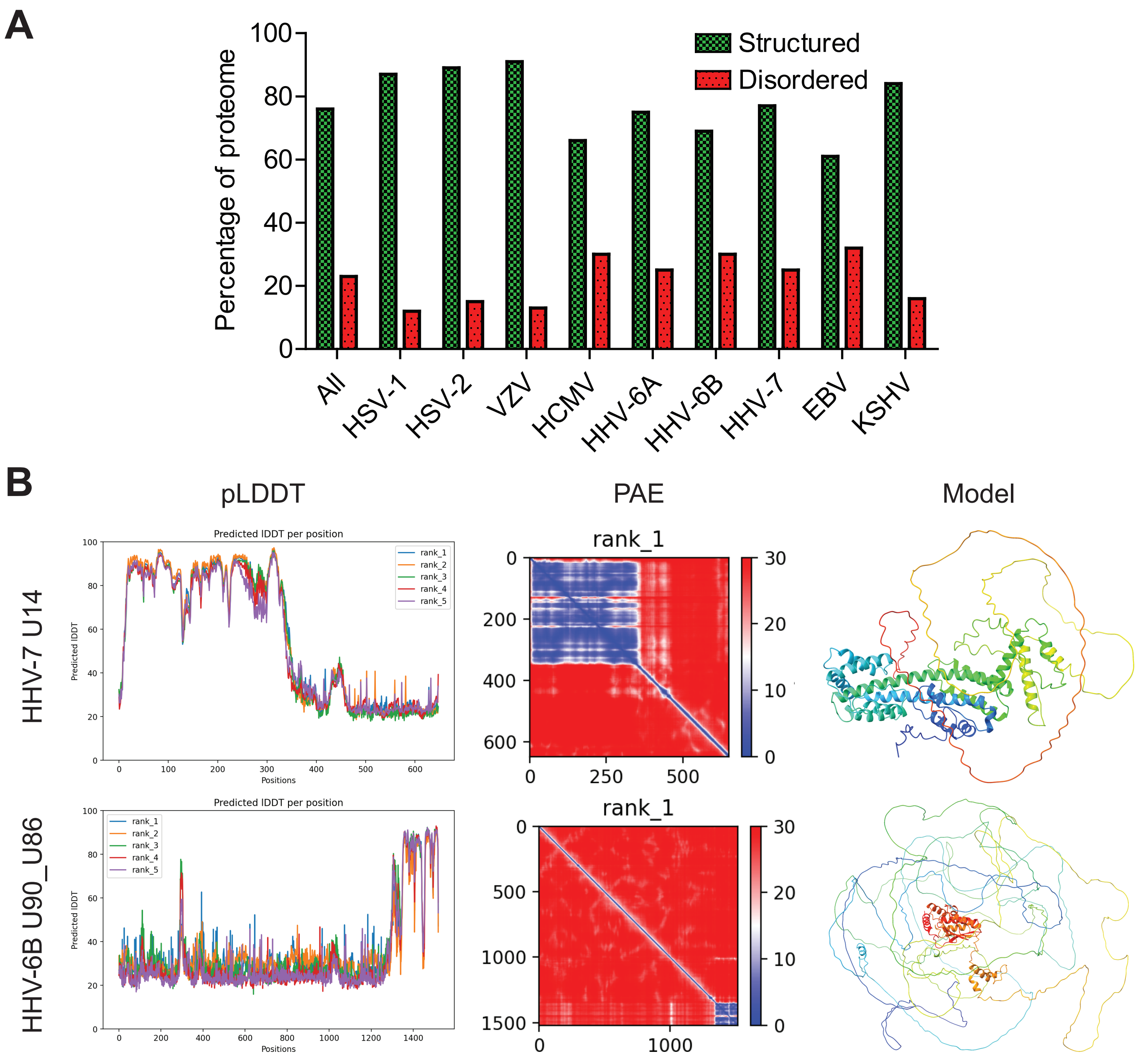

Supplementary Fig. 2. **Herpesviruses express a large fraction of proteins that contain a disordered domain.** A. The percentage of proteins that contain a structured or disordered domain are shown for each species as well as the consolidated proteome. B. HHV-7 U14 is an example of a protein with a structured N-terminus while HHV-6B U90_U86 has a structured C-terminus. The pLDDT and PAE plots are also shown to illustrate how the structured and disordered regions appear. The predicted structure is shown as a rainbow colored ribbon diagram where blue is the N-terminus and red is the C-terminus.

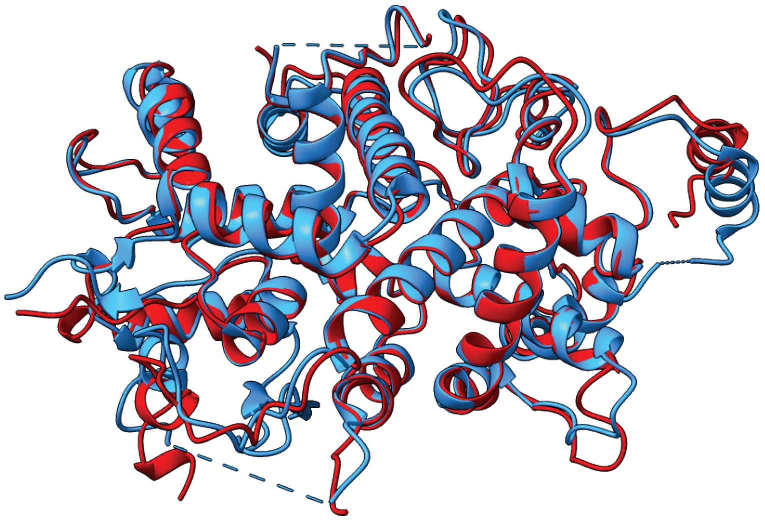

Supplementary Fig. 3. **Alignment of predicted HCMV UL77 structure to the experimental structure that was deposited post-AlphaFold training.** The deposited structure for HCMV UL77 (PDB 7NXP[19]) (blue) was aligned with the AlphaFold prediction (red), and the comparison has an RMSD of 0.509 Å against the 310 Cα. Only the residues present in the deposited structure are shown in the predicted model. The corresponding UCSF ChimeraX session can be found at https://zenodo.org/records/13284140.

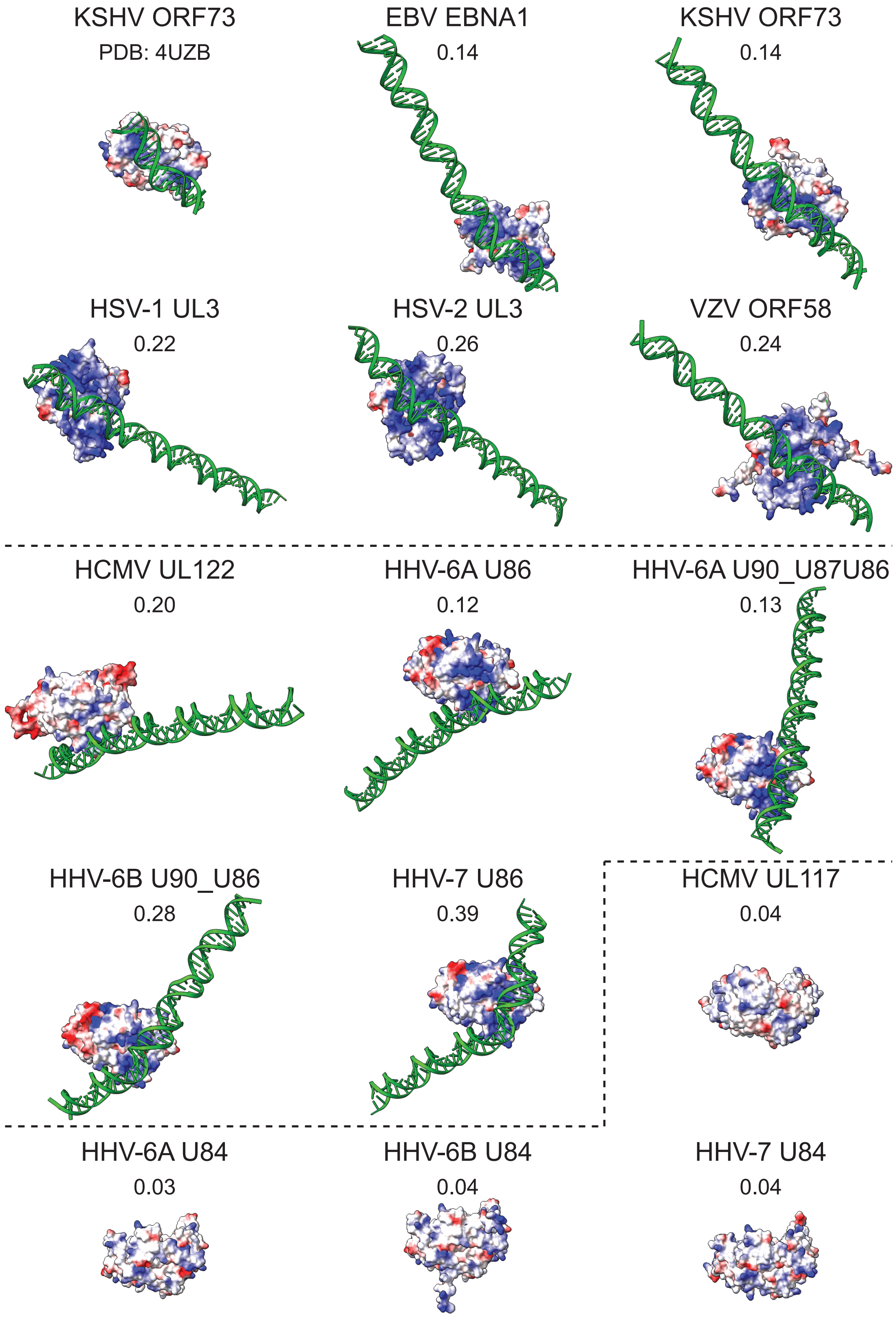

Supplementary Fig. 4. **Predicted positive surface charge and DNA binding activity of the KSHV ORF73 and EBV EBNA1 cluster.** The proteins in this cluster were structurally predicted as dimers of the conserved core with the DNA binding sequence of KSHV ORF73[80] with AlphaFold 3. The crystal structure of KSHV ORF73 with DNA (PDB: 4UZB[29]) was included for comparison. The average pairwise ipTM between each protein and DNA strand is shown above the structure. The dashed line separates the different binding phenotypes: KSHV ORF73, HCMV UL122 (different orientation), and HCMV UL117 (no binding). Positive surface charge is displayed as blue, and negative charge is displayed as red. The corresponding UCSF ChimeraX session can be found at https://zenodo.org/records/13284140.

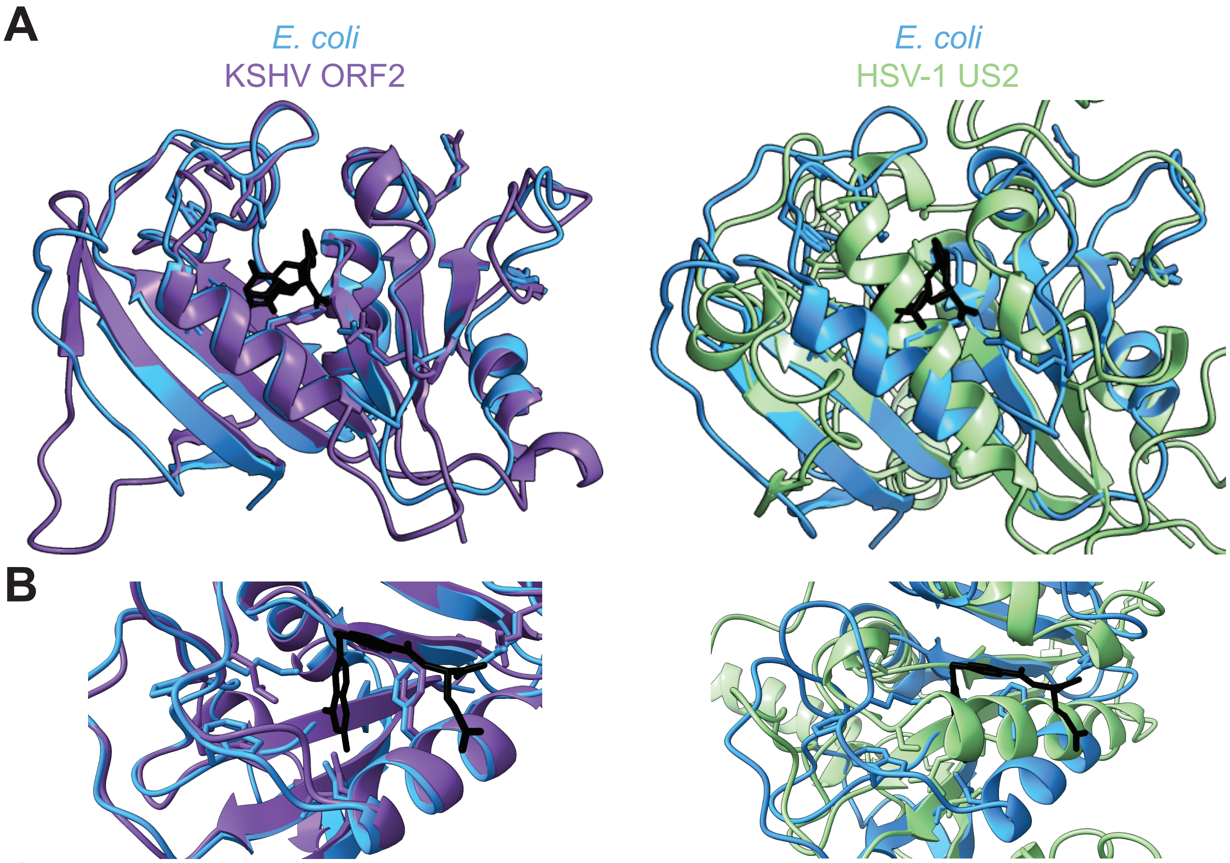

C

```
Structure based alignment

                                           ***         *  *
E. coli         .....MISLIAALAVD.RVI.GMENA.MPWN..L..PADLAWF.KRNTL. 36
KSHV_ORF2       .MDP.TLYCVVAVDTK.LGI.GKNRC.LPWP.AL..RGDMRRF.RQLTTD 41
HSV-1_US2       M.GVVVVNVMTLLDQNNALPRT..SVDA.S.PALWS...F..LLRQ.CRI 39
HSV-2_US2       M.GVVVVSVVTLLDQRNALPRT..SADA.S.PALWS...F..LLRQ.CRI 39
                      :  :. :                    *     :  : ::

                                 *                  *
E. coli         ..........DKPVIMGRHTWESIG....R..PLPGR...KNIILS.... 63
KSHV_ORF2       ..C.A..PGKQNMVVMGRRTWLSI.PAGCR..PLAGR...INVVLS.... 76
HSV-1_US2       LASEPLG...TPVVVR...PAN.LRR..LAEP.LMDLPKPTRPIVRTRSC 79
HSV-2_US2       LASEPLG...TPVVVR...PAN.LRR..LAEP.LMDLPKFTRPIVRTRSC 79
                             *:    .   :         * .     . ::

E. coli         .............SQ.PG.T.DDRV.TWVKS.VDEAI.AAC......... 85
KSHV_ORF2       .............RTLETPPP..GAHFLASS.LDAAL.GLAR..S.P..E 104
HSV-1_US2       RCPPNTTTGLFAE......DSPLES.TEVVDAVA.CFRL.LHRDQPSPP. 119
HSV-2_US2       RCPPNTTTGLFAE......DDPLES.IEILDAPA.CFRL.LHQERPGPH. 119
                                              .    .:

                             **                      *
E. coli         .GD...VPEIMVIGGGRVYEQFLP.K.....AQKLYLTHIDAEVEGDTHF 125
KSHV_ORF2       LA.QQID.KVWVIGGGNLYREALT.G..P.WPVRLFLTRVLHDFACDVFL 148
HSV-1_US2       .RL...Y.HLWVVGAADLCVPFLEYAQKIRLGVRFIAIKTPDAWVGEPW. 163
HSV-2_US2       .RL...Y.HLWVVGAADLCVPFFEYAQKTRLGFRFIATKTNDAWVGEPW. 163
                        .: *:*.. :    :          ::   :       :

E. coli         PD..YE.PDD.WESVFS......E...........FHDADAQNS.H..S 150
KSHV_ORF2       SHDSLA...AYARV.N.PKPGEQE...........RVFQ...ERGI..F 176
HSV-1_US2       AVP.TRFL..PEWT.VA......WTPFPAAPNHPLE....T..L...LSR 194
HSV-2_US2       PLP.DRFL..PERT.VS......WTPFPAAPNHPLE....N..L...LSR 194
                .

E. coli         YCFEILERR......................................... 159
KSHV_ORF2       YMFETYIKV.TQ...................................... 187
HSV-1_US2       YEYQYGVVLPGT.NGRE.RDC.MRWL.RSL..IALH.KPHPATP.GPL.T 235
HSV-2_US2       YEYQYGVVVPGDRERSCLR.WLR..SL...VAPH..NK.PRPASSR.PHP 234
                * ::

E. coli         ..................................................
KSHV_ORF2       ..................................................
HSV-1_US2       TSHPVRRPCCACMGMPEVPDEQPTSPGRGPQETDPLIAVRGERPR..... 280
HSV-2_US2       ATHPTQRPCFTCMG.............................RPEIP 253

E. coli         ..................................................
KSHV_ORF2       .............................................SSDTA 192
HSV-1_US2       ..............................LPHI.CYPVTTL........ 291
HSV-2_US2       DEPSWQTGDDDPQNPGPPLAVGDEWPPSSHV.CYPI......TNL..... 291

E. coli         ..................
KSHV_ORF2       LPDLERPRPATPPFSETS 210
HSV-1_US2       ..................
HSV-2_US2       ..................
```

```
CLUSTAL O(1.2.4)

                                      ***      *  *                      *
E. coli         ---MISLIAALAVDRVIGMENAMPWNLP-ADLAWFKRN----------TLNKPVIMGRHT 46
KSHV_ORF2       MDPTLYCVVAVDTKLGIGKNRCLPWPALRGDMRRFRQLTTDCAP----GKQNMVVMGRRT 56
HSV-1_US2       -----MGVVVVNVMTLLDQNNALPRTSVDASPALWSFLLRQCRILASEPLGTPVVVRPAN 55
HSV-2_US2       -----MGVVVVSVVTLLDQRNALPRTSADASPALWSFLLRQCRILASEPLGTPVVVRPAN 55
                       :..: .   :. ...:*     ..   :                . *::   .

                                    *
E. coli         W-------ESIG---RPLPGRKNIILSSQPGTDDRV---TWV-KS-------VDEA---- 81
KSHV_ORF2       W-------LSIPAGCRPLAGRINVVLSRTLETPPPGA--HFLASS-------LDAA---L 97
HSV-1_US2       LRRLAEPLMDLPKPTRPI------VRTRSCRCPPNTTTGLFAEDSPLESTEVVDAVACFR 109
HSV-2_US2       LRRLAEPLMDLPKFTRPI------VRTRSCRCPPNTTTGLFAEDDPLESIEILDAPACFR 109
                         .:    **:      : :             :  ..       :*

                                  **                  *
E. coli         -----IAACGDVPEIMVIGGGRVYEQFLPKA--QKLYLTHIDA----EVEG------DTH 124
KSHV_ORF2       GLARSPELAQQIDKVWVIGGGNLYREALTGPWPVRLFLTRVLH----DFAC------DVF 147
HSV-1_US2       LLHRDQPSPPRLYHLWVVGAADLCVPFLEYAQKIRLGVRFIAIKTPDAWVGEPWAVPTRF 169
HSV-2_US2       LLHQERPGPHRLYHLWVVGAADLCVPFFEYAQKTRLGFRFIATKTNDAWVGEPWPLPDRF 169
                           : .: *:*.. :    :      :* .  :                  .

E. coli         FPDY----------EPD-DWESVFSEFH-------DADAQNSHSYCFEILERR------- 159
KSHV_ORF2       LSHDSLAAYARVNPKPG-EQERVFQE--------------RGIFYMFETYIKVTQSSDTA 192
HSV-1_US2       LPEWTVA-WTPFPAAPNHPLETLLSRYEYQYGVVLPGTNGR-ERDCMRWLRSLIALH-KP 226
HSV-2_US2       LPERTVS-WTPFPAAPNHPLENLLSRYEYQYGVVVPGD--R-ERSCLRWLRSLVAPHNKP 225
                : .            *.   * ::..              .     :.

E. coli         ------------------------------------------------------------ 159
KSHV_ORF2       LPDLERPRPATPPFSETS------------------------------------------ 210
HSV-1_US2       HPATPGPLTTSHPVRRPCCACMGMPEVPDEQPTSPG-RGPQETDPLIAVRGERPRLPHIC 285
HSV-2_US2       RPASSRPHPATHPTQRPCFTCMGRPEIPDEPSWQTGDDDPQNPGPPLAVGDEWPPSSHVC 285

E. coli         ------         159
KSHV_ORF2       ------         210
HSV-1_US2       YPVTTL         291
HSV-2_US2       YPITNL         291
```

Supplementary Fig. 5. **Alignment of E. coli DHFR to KSHV ORF2 and HSV-1 US2.** A. The *E. coli* DHFR (PDB 1RX2[48]) (blue) was aligned to KSHV ORF2 (purple) and HSV-1 US2 (green), and the cofactor folic acid is shown in black. The atomic structure of the catalytic residues of the *E. coli* DHFR (M20, P21, W22, D27, F31, R44, R57, G95, G96, T113) and the potential catalytic residues that are in similar positions in ORF2 and US2 are shown. B. An enlarged view of the catalytic site is shown. C. The protein sequences were aligned based on the structure-based alignment or with Clustal Omega. The *E. coli* catalytic residues are marked with red asterisks. The potential catalytic residues in the herpesvirus proteins are from the structured based alignment. The corresponding UCSF ChimeraX session can be found at https://zenodo.org/records/13284140.

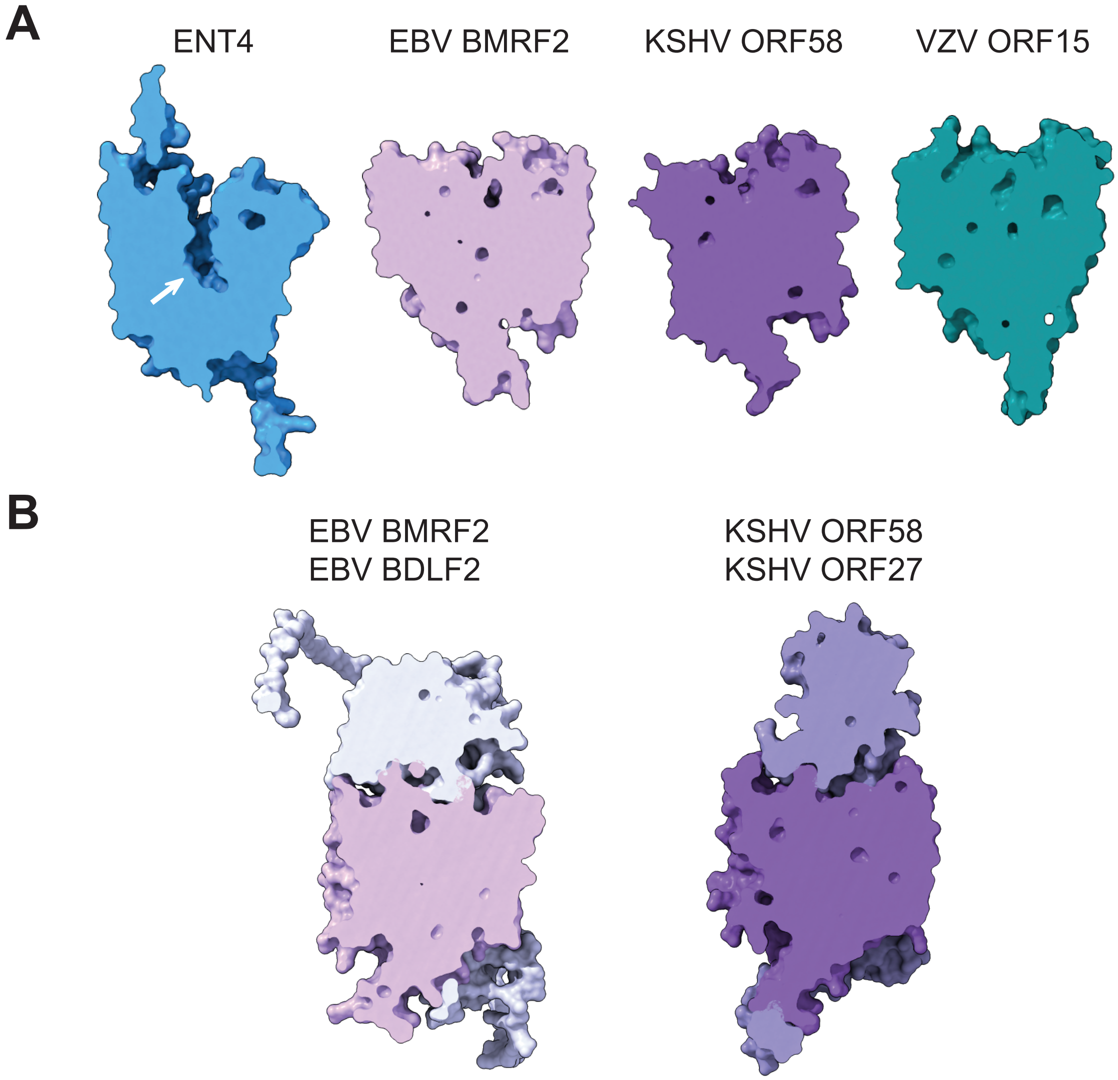

Supplementary Fig. 6. **The herpesvirus ENTs are unlikely to have transporter activity.** A. Cross-sections of surface plots of the monomer predictions of ENT4 (AF-Q8R139-F1-v4) and the structurally similar viral proteins. The white arrow points to the metabolite channel in ENT4. B. Structure predictions of the known EBV (BMRF2 pink and BDLF2 white) and KSHV (ORF58 purple and ORF27 light purple) heterodimers. The corresponding UCSF ChimeraX sessions can be found at https://zenodo.org/records/13284140.
