# Supplementary material for: A proteome-wide structural systems approach reveals insights into protein families of all human herpesviruses
Source: Nat Commun. 2024 Nov 26;15:10230. doi: 10.1038/s41467-024-54668-2 (PMC11599850; doi:10.1038/s41467-024-54668-2)
Supplement: Supplementary file 3 — Description Of Additional Supplementary File [file 41467_2024_54668_MOESM3_ESM.pdf]

Description of Additional supplementary files

Supplementary Data 1. Consistency of pLDDT scores of AlphaFold models.

Description: The quality scores for LocalColabFold with 3x recycles are in Sheet 1, AlphaFold are in Sheet 2, and LocalColabFold with 20x recycles are in Sheet 3. Sheet 4 lists all proteins and details whether they passed the quality scores. Sheet 5 lists the percentages of each protein sequence with a pLDDT of 0.70 (likely structured). Sheet 6 lists the DeepTMHMM topology prediction of all proteins.

Supplementary Data 2. Identified herpesvirus structural similarity clusters.

Description: Sheet 1 lists the currently known homologous proteins between herpesvirus species and the Foldseek outputs. Sheet 2 shows the clustering of the herpesvirus proteins using the sequence alignment-based algorithm MMseqs2, and Sheet 3 shows the clustering using the sequence HMM-HMM based algorithm HHblits. Sheet 4 lists the newly identified structural similarity groups based on DALI analysis with reference to the previously known homolog groups shown in Sheet 1. The identified function based on structural similarity to cellular structures is annotated. Sheet 5 is the same as Sheet 4, except that Foldseek outputs were used for clustering by structural similarity. Sheet 6 lists the structural clusters based on domain-level search instead of full-length protein search. The difference to the full-length analysis is annotated. Sheet 7 shows the evaluation of the different Foldseek parameters. Specifically, it compares the different alignment algorithms, significance metrics, and significance thresholds. Sheet 8 compares the differences between analyzing full length herpesvirus proteins with Foldseek using the E-value versus the probability. The differences are highlighted. Sheet 9 compares the difference between analysing full-length proteins and proteins truncated based on the pLDDT score with Foldseek. The differences are highlighted. Sheet 10 lists all output protein pairs from HHblits and Foldseek along with the significance scores. A “-” denotes that the protein pair was not exported by the algorithm. For the significant pairs, they were classified as significant with only HHblits, with only Foldseek, or with both. Each protein pair has a score for protein A against B as well as B against A.

Supplementary Data 3. Results of the domain-level structural similarity search.

Description: Sheet 1 lists all pairwise similarities found using Foldseek for full-length similarity searches (columns AB) and domain-level searches using structural snippets generated by the sliding window approach (columns C-D) for comparison. Pairs only identified in the domain-level search are listed again (columns E-F). Sheet 2 is a list of the sequence ranges of query proteins that match the sequence ranges of a target protein in the domain-level search. Sheet 3 lists all domain-level search results as well as a categorization of whether the hits can be categorized into three categories: "Internal Duplication" (the query protein matched itself), "Repetitive Acquisition" (the query protein matched a target sequence multiple times), or "Domain Addition" (the query protein has a domain that is not found in a related protein).

Supplementary Data 4. Structural similarity search against cellular proteins.

Description: Sheet 1 lists the Foldseek search results of the viral protein structure predictions against the PDB database structural similarity search. Sheet 2 lists the frequency of each word in the description of the hits from sheet 1 for each protein (column B). Viral keywords were subtracted to remove known

functions and self-hits from experimental viral structures in the PDB (column C). Sheet 3 lists the Foldseek structural similarity search results of the viral protein structure predictions against the AlphaFold-SwissProt database. Sheet 4 lists the frequency of each word in the description of the hits from sheet 3 for each protein (column B). Viral keywords were subtracted to remove known functions and self-hits from structures in the AlphaFold-Swiss-Prot database (column C). Sheet 5 lists which herpes proteins had a structurally similar herpes (column A) or cellular (column B) protein or is structurally unique (column C). It also lists viral clusters for which the whole cluster did not have any structurally similar proteins in the AlphaFold-Swiss-Prot database (column D). The PDB database was left out of the comparison for this sheet because the PDB also contains experimentally solved herpesvirus structures.
